# Cost-Effective Modular Biosensor for SARS-CoV-2 and Influenza A Detection

**DOI:** 10.3390/bios13090874

**Published:** 2023-09-07

**Authors:** Andrew Murray, Julio Ojeda, Omar El Merhebi, Percy Calvo-Marzal, Yulia Gerasimova, Karin Chumbimuni-Torres

**Affiliations:** Department of Chemistry, University of Central Florida, Orlando, FL 32816, USA; murrayand@knights.ucf.edu (A.M.); juliohector.ojedavelarde@ucf.edu (J.O.); merhebi.omar@outlook.com (O.E.M.); percy.calvomarzal@ucf.edu (P.C.-M.); yulia.gerasimova@ucf.edu (Y.G.)

**Keywords:** four-way junction, SARS-CoV-2, Influenza A, biosensor, microliter detection

## Abstract

A modular, multi-purpose, and cost-effective electrochemical biosensor based on a five-stranded four-way junction (5S-4WJ) system was developed for SARS-CoV-2 (genes S and N) and Influenza A virus (gene M) detection. The 5S-4WJ structure consists of an electrode-immobilized universal stem-loop (USL) strand, two auxiliary DNA strands, and a universal methylene blue redox strand (UMeB). This design allows for the detection of specific nucleic acid sequences using square wave voltammetry (SWV). The sequence-specific auxiliary DNA strands (m and f) ensure selectivity of the biosensor for target recognition utilizing the same USL and UMeB components. An important feature of this biosensor is the ability to reuse the USL-modified electrodes to detect the same or alternative targets in new samples. This is accomplished by a simple procedure involving rinsing the electrodes with water to disrupt the 5S-4WJ structure and subsequent re-hybridization of the USL strand with the appropriate set of strands for a new analysis. The biosensor exhibited minimal loss in signal after rehybridization, demonstrating its potential as a viable multiplex assay for both current and future pathogens, with a low limit of quantification (LOQ) of as low as 17 pM.

## 1. Introduction

At the beginning of the COVID-19 pandemic in 2019, identifying carriers of severe acute respiratory syndrome coronavirus 2 (SARS-CoV-2) was crucial for containing the virus and mitigating the initial impact of the pandemic [1,2]. Prior to the availability of point-of-care (POC) and at-home antigen tests, nucleic acid testing (NAT) using reverse transcription quantitative polymerase chain reaction (RT-qPCR) was the primary method for testing SARS-CoV-2 [2]. Despite being the gold standard technique for genomic testing of many viral infections, qRT-qPCR assays presented several drawbacks that limited their suitability for rapid response during the early stages of a pandemic [1,2]. These assays typically have slow turnaround times due to the need for highly trained personnel and centralized laboratory settings [1]. Additionally, their high cost renders RT-qPCR assays unavailable in many low- and middle-income countries, which have been disproportionately impacted by the pandemic [3,4,5]. Therefore, these tests are not an effective approach to tracing a pathogen, particularly at the immediate onset of a pandemic, when assay speed and accessibility are paramount.

Conversely, rapid testing administered at POC settings, such as rapid antigen tests and NATs, has significantly contributed to the reduction of SARS-CoV-2 cases [1,6,7,8,9]. Tests conducted at POC settings eliminate the need for sample storage and transport, and results can usually be provided to the patient on the same day as sample collection. However, such tests were not immediately available at the beginning of the COVID-19 pandemic. The first POC test for SARS-CoV-2, the Cepheid Xpert Xpress SARS-CoV-2 assay, was authorized by the Food and Drug Administration (FDA) in March 2020 [10,11]. Therefore, rapid development of assays for new pathogens during future pandemics may prove essential to containing emerging viruses.

The emergence of different variants of concern (VOC) of SARS-CoV-2, resulting from mutations in the RNA genome, has posed challenges for many testing platforms, including serological and RT-qPCR-based methods [12,13,14,15,16,17]. NAT-based assays can be quickly adapted to target multiple variants in one reaction by using appropriate primer and probe combinations [18,19]. However, adapting antigen tests for new variants may be more difficult, as existing tests may require redesigning or the development of new variant-specific antigen tests [15,18,20]. Therefore, while antigen tests are commonly used in POC diagnostics, NAT-based assays continue to demonstrate superiority in terms of versatility and adaptability to emerging variants. This is reflected in the great number of NAT-based platforms that were develop since then for POC, like the Visby Medical RT-PCR portable device, the Cue COVID-19 test, the ID NOW COVID-19 assay, the Accula SARS-CoV-2 lateral flow, etc., all of which target the N gene or RdRp gene [21].

These factors highlight the need for a new niche in testing: assays that can be rapidly developed, manufactured, and distributed at the onset of a pandemic while being cost-effective and suitable for POC settings. This approach would facilitate a rapid response and effective containment of novel pathogens or pathogen variants [12]. Biosensing platforms, particularly DNA biosensors using electrochemical detection (E-biosensors), are well-suited for this role [10,22]. Such E-biosensors have demonstrated high sensitivity and selectivity in detecting nucleic acids while also being portable and easy to use [10,22,23,24].

We and others have previously reported an E-biosensor based on the formation of a four-way junction (4WJ) complex [10,25,26,27,28,29,30]. The 4WJ complex consists of a universal stem-loop (USL) strand and two auxiliary DNA strands: f and m. These auxiliary DNA strands are designed to have complementary fragments with both the target nucleic acid of interest and the USL, enabling the formation of a four-strand complex. This allows the approach of a redox-active marker to the electrode’s surface. The E-biosensor exhibits high selectivity, as both auxiliary DNA strands must be bound to the target simultaneously for the signal to appear.

**Figure 1:**
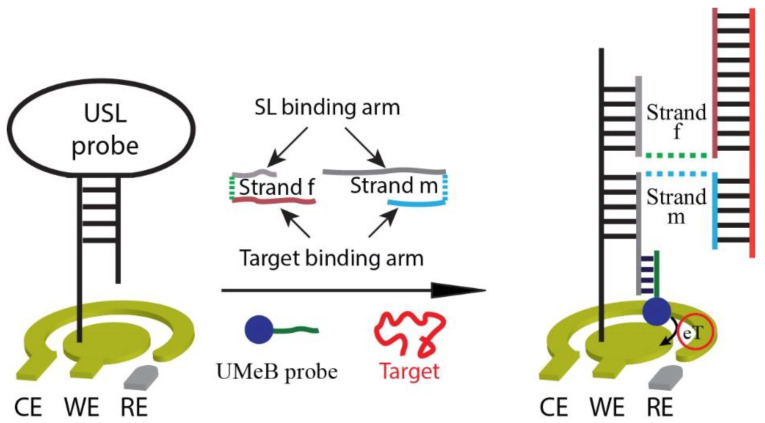
Scheme of the 5S-4WJ-based biosensor.

While the 4WJ biosensor shows promise for virus detection in resource-limited settings and POC applications, standardizing the most expensive component, the MeB redox marker conventionally conjugated to the m-strand, minimizes the cost associated with analyzing different viruses. In this work, we utilized a universal sequence attached to the MeB redox marker, creating a universal MeB (UMeB) probe and forming the five-stranded (5S)-4WJ structure (Figure 1). The ability to re-use the most expensive components of the 4WJ-based E-biosensor, he USL strand, which we have previously demonstrated [29], and the MeB-containing strand [25] helps reduce the costs associated with the sensor assay and allows the detection of multiple viruses, this being an important feature in diagnostic scenarios when multiple nucleic acid targets need to be analyzed. By utilizing isothermal nucleic acid sequence-based amplification (NASBA) and disposable screen-printed gold electrodes (SPGEs), the biosensor can serve as a cost-effective diagnostic assay for detecting multiple RNAs. Furthermore, since the E-biosensor employs universal DNA probes (USL and UMeB), it can be easily modified to detect different viruses, including new viruses or virus variants, without the need for complete biosensor re-manufacturing. Additionally, the E-biosensor can be regenerated through a simple washing step at room temperature and reused for the detection of other target sequences. Therefore, this E-biosensor holds significant potential as a versatile, cost-effective, and user-friendly diagnostic platform for future pandemics, particularly in POC settings.

## 2. Materials and Methods

### 2.1. Materials and Reagents

The 2019-nCoV_N_Positive Control, 2019-nCoV RUO Primer/Probe Kit, and oligonucleotides for NASBA primers, biosensor adaptor strands, UMeB redox probe, and synthetic target fragments were purchased from Integrated DNA Technologies (Coralville, IA, USA). Sequences of all oligonucleotides are listed in Table S1. A NASBA kit was obtained from Amsbio LLC (Cambridge, MA, USA). Genomic RNA from SARS-related coronavirus 2 was obtained through BEI Resources (Manassas, VA, USA). This reagent was deposited by the Centers for Disease Control and Prevention and obtained through BEI Resources, NIAID, NIH: genomic RNA from SARS-related coronavirus 2, isolate USA-WA1/2020, NR-52285. Agarose powder and SYBR Safe 10,000X stain were obtained from Thermo Fisher Scientific (Waltham, MA, USA). Trizma hydrochloride (Tris-HCl), 6-mercapto-1-hexanol (MCH), MgCl_2_, and tris (2-carboxyethyl) phosphine hydrochloride (TCEP) were purchased from Sigma-Aldrich (St. Louis, MO, USA). Sulfuric acid, NaCl, and NaOH were purchased from Fisher Scientific (Pittsburgh, PA, USA). Gold disk electrodes (GDEs) were purchased from CH Instruments (Austin, TX, USA). Screen-printed gold electrodes (SPGEs) (C220BT) were purchased from Metrohm DropSens (Riverview, FL, USA). Alumina slurry was obtained from Buehler (Lake Bluff, IL, USA). The immobilization buffer (IB) was prepared with 250 mM NaCl and 50 mM Tris-HCl and adjusted to a pH of 7.4 using 1.0 M NaOH. The hybridization buffer (HB) was prepared with 100 mM NaCl, 50 mM Tris-HCl, and 50 mM MgCl_2_, and adjusted to a pH of 7.4 using 1.0 M NaOH.

### 2.2. NASBA Reaction

Genomic RNA from SARS-related coronavirus 2, isolate USA-WA1/2020 (BEI Resources), was used as a template for NASBA of the viral S gene. A series of template dilutions (50-fold, 150-fold, 500-fold, 1500-fold, or 5000-fold) was prepared and quantified by following the protocol approved by the CDC for 2019-nCoV RT-qPCR using the StepOnePlus™ Real-Time PCR System (ThermoFisher Scientific, Waltham, MA, USA). The 2019-nCoV RUO Primer/Probe Kit and a calibration curve obtained with the 2019-nCoV_N_Positive Control in the same RT-qPCR analysis. The analysis was performed in triplicate, and the determined concentration was averaged to be (1.4 ± 0.1) × 10^4^ copies/μL, (4.9 ± 0.6) × 10^3^ copies/μL, (2.0 ± 0.1) × 10^3^ copies/μL, (6.9 ± 0.6) × 10^2^ copies/μL, and 236 ± 16 copies/μL for 50-fold, 150-fold, 500-fold, 1500-fold, or 5000-fold template dilutions, respectively. The same dilutions were used for the same-day NASBA reaction using a NASBA Wet Kit, according to the vendor-recommended protocol, with slight modifications. Specifically, the samples containing the template (2 μL), reaction buffer (4 μL of the 3 × stock), primers forward and reverse (2 µL of a mixture, with each primer at 1.5 µM), and a mixture of dNTPs/NTPs (2 μL of the 6 × stock) were incubated at 65 °C for 2 min, then at 41 °C for 10 min, followed by addition of the enzyme mixture (3 μL of the 4 × stock) and incubation at 41 °C for 90 min. For the no-template control (NTC), RNase-free water (2 μL) was used instead of the viral RNA. The amplicons were analyzed via gel electrophoresis on a 2% agarose gel containing Gel Red dye and visualized using a Bio-Rad Gel Doc XR+ with Image Lab software.

### 2.3. Electrochemical Measurements

Electrochemical measurements, such as square-wave voltammetry (SWV) and cyclic voltammetry (CV), were performed on a CHI660D electrochemical workstation. For GDE, a conventional three-electrode configuration was used, which consisted of a GDE as a working electrode, a platinum wire as an auxiliary electrode, and a silver/silver chloride (Ag/AgCl) reference electrode. The SPGEs included a three-electrode setup: a gold working electrode, a gold reference electrode, and a silver reference electrode. SWV measurements were performed in HB (10 mL for GDEs or 100 μL for SPGEs) from 0.0 to −0.5 V with a 100 Hz frequency, 70 mV amplitude, and a 3 mV step potential. The HB solution was bubbled with nitrogen for 10 min to remove all dissolved oxygen before measurements were started. Measurements were performed in triplicate at 25 °C.

### 2.4. Biosensor Preparation

GDEs were first cleaned by placing them in a piranha solution (1:3 ratio of 30% H_2_O_2_ to 95–98% H_2_SO_4_; caution: the mixture of H_2_O_2_ and H_2_SO_4_ is highly corrosive and should be handled with care) for 10 min and then washing rigorously. GDEs were then polished with a microcloth with 1.0, 0.3, and 0.05 μm alumina slurry and sonicated in ethanol and water for 2 min each to remove residual alumina particles. The GDEs were then activated via CV in 0.5 M H_2_SO_4_ from 1.6 to −0.1 V for 10 cycles at a scan rate of 100 mV/s. The electrochemically active area was calculated using the gold reduction peak from CV.

SPGEs were activated by drop-casting 100 μL of 0.5 M H_2_SO_4_ onto the surface, followed by performing CV from 1.2 to −0.1 V for 10 cycles at a scan rate of 100 mV/s. The electrochemically active area was again calculated using the gold reduction peak from CV.

After the initial cleaning/activation of the electrode, the process of preparing the GDEs and SPGEs was the same. The universal stem-loop (USL) strand was first treated with TCEP (1 mM), with shaking at 25 °C for 1 h, to ensure a reduced state of the thiol group. The USL solution was then diluted to 0.1 μM using IB, and 15 μL of this solution was drop-casted onto each electrode and incubated for 30 min at 25 °C. Next, the electrodes were rinsed with IB to remove the excess of the unbound USL strand and dried with nitrogen. Then, 15 µL of 2 mM MCH in IB was drop-casted onto each electrode and incubated for 30 min at 25 °C to avoid non-specific absorptions onto the gold surface. Then, the electrodes were rinsed with IB and dried with nitrogen. Prior to hybridization of the biosensor with the target, a baseline signal was established using SWV (described above).

For the formation of the 5S-4WJ structure, a hybridization solution containing 0.5 μM f-strand, 0.1 μM m-strand, 0.25 μM UMeB probe, and varying concentrations of DNA target or 10% (*v*/*v*) NASBA amplicon in HB was prepared. 15 μL of this solution was drop-cast onto each electrode and allowed to incubate for 10 or 30 min at 25 °C. The electrode was then rinsed with HB before testing. The electrode’s response is expressed as the current density peak (j_p_) from SWV. The current density peak was calculated by subtracting the baseline current and dividing by electrochemically active area of the electrode.

### 2.5. Biosensor Characterization

Initial optimization and characterization of the biosensor were performed using a synthetic DNA target SARS-S corresponding to the analyzed fragment of the SARS-CoV-2 S-gene (Table S1). The biosensor was prepared on a GDE with a 50 nM DNA target, and the hybridization time was optimized by evaluating the signal after 1-, 5-, 10-, 15-, 30-, 45-, and 60-min hybridization. 

Calibration curves for 3 different targets: SARS-S, SARS-N (corresponding to a fragment of SARS-CoV-2 gene N), and InfA-M (corresponding to a fragment of the Influenza A virus gene M) were constructed for the GDE-immobilized E-biosensors at 10 min of hybridization time. Additionally, for SARS-S, two calibration curves were constructed using SPGEs instead of GDEs at 10 and 30 min of hybridization time, all using the 5 to 50 nM target concentration range. Limit of detection (LOD) and limit of quantification (LOQ) were calculated as 3.3 σ/s and 10 σ/s, respectively, where σ is the standard deviation from the blank measurement and s is the slope of the respective calibration curve.

### 2.6. Study of Biosensor Reusability and Selectivity

The re-usability of the USL-modified GDE was tested by immersing the electrode with the complete 5S-4WJ structure attached in 40 mL of deionized water with agitation for 5 min at 25 °C to disassemble the 5S-4WJ structure, leaving the USL probe on the electrode’s surface available to use in a new analysis. After drying the USL-GDE with nitrogen, a new hybridization solution containing 5S-4WJ components was cast onto the electrode’s surface, and the above-mentioned procedure for the formation of the 5S-4WJ structure on the electrode was followed.

For selectivity testing, different hybridization solutions were prepared. First, the m- and f-strands for InfA-M and SARS-S were tested, each with their respective targets at 50 nM. Additionally, the m- and f-strands for InfA-M were tested against the SARS-S target, and the m- and f-strands for SARS-S were tested against InfA-M.

## 3. Results

### 3.1. Design of the Electrochemical E-Biosensor

The E-biosensor consists of five DNA strands, one of which is the universal stem-loop (USL) strand. The USL is covalently bound to the surface of a gold electrode through its terminal thiol group. To prevent the absorption of non-specific compounds, a monolayer of 6-mercapto-1-hexanol (MCH) is also attached. The USL acts as a tether, connecting a specific target to the electrode by forming a five-stranded, four-way junction (5S-4WJ) structure using two auxiliary strands: m and f, as depicted in Figure 1. These auxiliary strands have complementary fragments for both the USL and the target. This design follows the concept of multicomponent (split or binary) hybridization probes [31], where a signal is reported, only both target-binding fragments of m- and f-strands need to be fully complementary to the target to elicit a signal. As a result, one auxiliary strand can be made short enough to ensure the differentiation of targets with single-nucleotide substitutions. At the same time, for successful interrogation of folded nucleic acid targets, the target-binding fragment of the second auxiliary strand is made longer [32], thus enabling both high probe-target affinity and selectivity. In our design, the f-strand plays a role in unwinding the target’s secondary structure and enabling efficient 5S-4WJ formation, while the m-strand contributes to the high selectivity of target recognition. 

In addition to USL, the 5S-4WJ incorporates another universal, target-independent strand: the UMeB strand [25]. UMeB contains a MeB redox indicator and is complementary to a fragment of the m-strand adjacent to its USL-binding fragment. Consequently, the presence of the target leads to the formation of the target-dependent 5S-4WJ structure at the electrode’s surface, resulting in an increase in current, which is detected due to the presence of the MeB indicator on the UMeB strand (Figure 1).

### 3.2. Biosensor Response towards Fragments of SARS-CoV-2 Genes S and N, and Influenza A Virus Gene M 

In this work, the 5S-4WJ E-biosensor was designed and tested to detect genomic fragments of SARS-CoV-2 and Influenza A virus. Specifically, a fragment of the S gene from SARS-CoV-2 was chosen as a primary target for all optimization procedures. Additionally, since the platform utilizes target-independent universal USL and UMeB strands, it can be adapted to detect other targets with a simple adjustment of the target-binding arms of the m-strand and f-strand to the new target sequence, providing a multi-purpose E-biosensor. The sequences of the targets and components of the correspondent 5S-4WJ biosensors are listed in Table S1.

To optimize the performance of the 5S-4WJ biosensor, the time required for efficient hybridization of the biosensor components with the SARS-S target was first optimized. As depicted in Figure S1, the highest signal was achieved after a 60-min hybridization without evidence of current saturation. However, even 10 min was enough to differentiate the blank from the target-induced signal (Figure S1, inset). This enables shortening of the assay’s time. Therefore, a 10-min hybridization time was employed for subsequent experiments.

**Figure 2:**
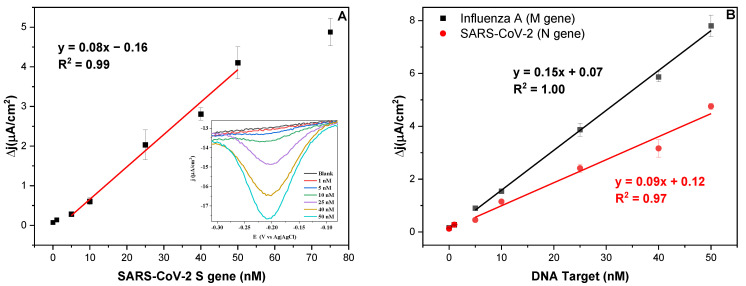
(**A**) Biosensor’s response towards varied concentrations (0, 1, 5, 10, 25, 40, 50, and 75 nM) of SARS-S target with a 10-min hybridization time on GDEs. Inset: Corresponding SWV for each of the concentrations tested. (**B**) Calibration curve for SARS N and InfA-M targets, both with a 10-min hybridization time on GDEs.

The dependence of the biosensor’s response on the target concentration (0–75 nM) was tested using GDE_S_ with the SARS-S DNA target (Table S1). As seen in Figure 2A, the best linear fit was observed in the range of 5–50 nM (R^2^ = 0.99), showing a current saturation around 75 nM. The LOD and LOQ were determined to be 0.19 nM and 0.57 nM, respectively. The same linear dynamic range of 5–50 nM was observed for the biosensors targeting both SARS-N and InfA-M (R^2^ = 0.97 and 1.00, respectively) (Figure 2B). Their LOD and LOQ were determined to be 0.49 nM and 1.49 nM, respectively, for SARS-N and 0.22 nM and 0.65 nM, respectively, for InfA-M.

Both biosensors targeting SARS-N and SARS-S targets showed similar analytical sensitivity, with slopes of 0.09 and 0.08 µA/(cm^2^ × nM), respectively. For the biosensor targeting InfA-M, a slope of 0.15 µA/(cm^2^ × nM) was obtained.

The performance of the SARS-S biosensor was also tested on SPGEs at two different hybridization times: 10 and 30 min (Figure S2). Unlike conventional GDEs, SPGEs require only microliters of a sample (for measurements), and no polishing treatment is needed before the electrode can be immobilized with the USL strand, reducing the procedure time. Therefore, SPGEs offer an advantage over GDEs, especially when multiple tests need to be performed on the same sample. Nevertheless, the LOD and LOQ for the 10 min hybridization time system were determined to be 0.30 nM and 0.92 nM, respectively. These values are slightly higher than the ones using GDEs under the same conditions. Therefore, GDEs were used for the subsequent experiments. Once the hybridization time was increased to 30 min on SPGEs, the sensitivity increased from 0.09 to 0.34 µA/(cm^2^ × nM). The new LOD and LOQ under these conditions were 5.82 and 17.63 pM, respectively, which corresponds to an approximately 50-fold improvement in LOQ at the expense of a slightly longer assay time.

### 3.3. Biosensor Reusability and Selectivity

**Figure 3:**
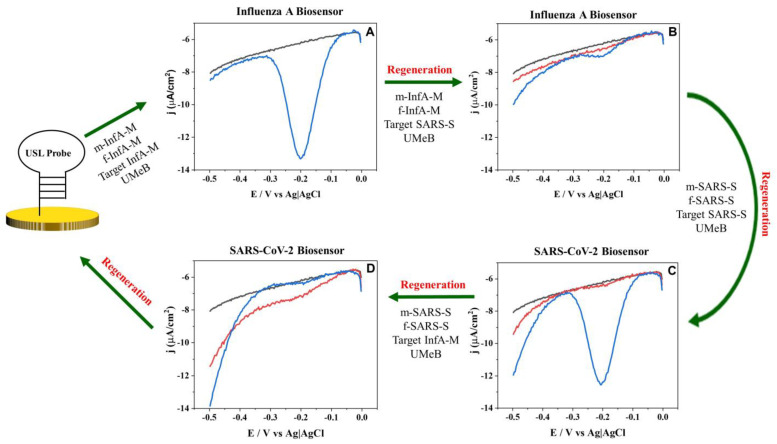
Regeneration and rehybridization of the USL probe for recognition of InfA-M and SARS-S. Square wave voltammetry response after hybridization with (**A**) m-InfA-M, f-InfA-M, InfA-M target and UMeB, (**B**) m-InfA-M, f-InfA-M, SARS-S target and UMeB, (**C**) m-SARS-S, f-SARS-S, SARS-S target and UMeB, (**D**) m-SARS-S, f-SARS-S, InfA-M target and UMeB on GDE. Black lines depict the baseline signal; blue lines depict the signal after hybridization or rehybridization of the USL-modified GDE with the indicated strands; red lines depict the signal after washing the 5S-4WJ components from the electrode (regeneration).

Due to the universal nature of the USL, it would be attractive to re-use the USL-modified electrodes for multiple rounds of analysis or for testing the same biosample for different pathogens. Therefore, we tested the feasibility of removing all but the USL component of the 5S-4WJ structure from the electrode’s surface for subsequent re-hybridization of the electrode-bound USL with new strands. As can be seen in Figure 3A, the InfA-M-specific biosensor produces the expected response in the presence of InfA-M (Figure 3A, blue curve), but upon rinsing the electrode with water, the signal goes back to the background level (Figure 3B, black curve). Moreover, the USL could be regenerated and rehybridized with the SARS-S-specific strands without any difference in the target-induced electrochemical signal (Figure 3C, blue curve). Furthermore, it was shown that the auxiliary strands for both SARS-S and InfA-M were specific to their respective targets, as the signal was only produced when target-specific m- and f-strands were used (compared to the blue curves for Figure 3A–D). These results demonstrate that the same E-biosensor can be used to test for both SARS-S and InfA-M by just switching the adaptor strands, which enables selective interrogation of the viral targets. Such discrimination is very important to facilitate the clinical diagnosis of these viral diseases due to the similarity of SARS-CoV-2 and Influenza A symptoms. 

### 3.4. Biosensor Response for SARS-CoV-2 RNA

Based on the lowest LOD and LOQ values obtained for the 5S-4WJ biosensors, it will be challenging to detect viral targets in samples with a low copy number viral load. Therefore, nucleic acid amplification is needed. In this work, to overcome the need for precise temperature control and complex thermocycling regimes, the NASBA technique was chosen. This technique offers the ability to isothermally amplify single-stranded RNA (ssRNA) [33]. 

The primers for the NASBA reaction were designed to amplify a fragment (nts 24077–24195) of the SARS-CoV2, isolate USA-WA1/2020, genome (GenBank: MT246667). The amplicon of 128 nts (including 9 nts transcribed from the forward primer downstream from the promotor sequence) was generated as expected (Figure S3). The sequences of the designed primers and expected amplicons are listed in Table S1.

**Figure 4:**
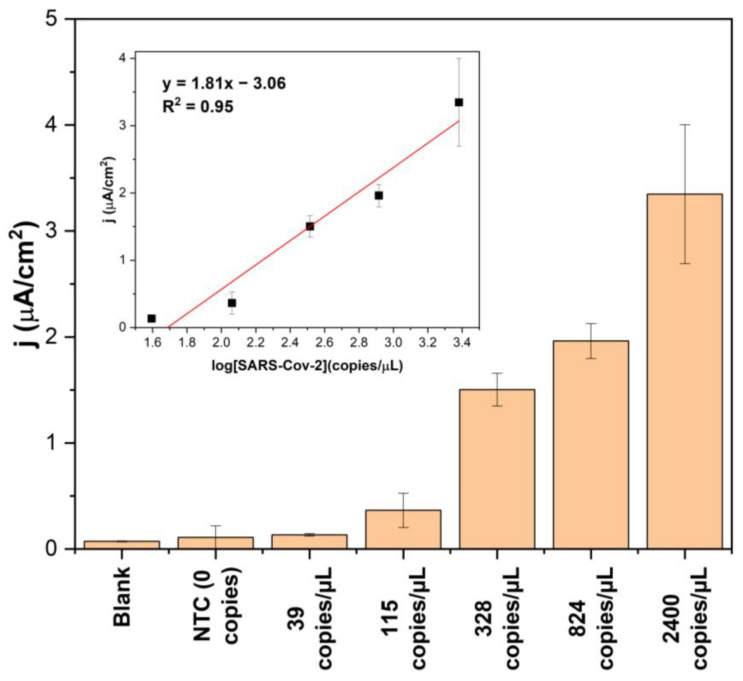
Response of the SARS-CoV-2 biosensor to the samples from NASBA (10% *v*/*v*). NTC: NASBA no-template control. Inset: dependence of the biosensor response on the logarithm of SARS-CoV-2 RNA concentration taken for the amplification step.

The developed electrochemical platform was tested with RNA NASBA amplicons obtained by amplifying different dilutions of total viral RNA that ranged from 39 copies/µL to approximately 2400 copies/µL. The NASBA samples were tested using the SARS-S-specific biosensor without the need for amplicon purification. The current density triggered by the NASBA no-template control (NTC) did not exceed the blank signal (Figure 4). The lowest SARS-S RNA dilution that could be reliably differentiated from the NTC after amplification was 115 copies/µL. The signal exhibits a linear dependence (with an R^2^ value of 0.95) on the logarithm of the RNA concentration taken for the NASBA reaction (Figure 4, inset), which is consistent with the exponential amplification mode of the amplification technique.

## 4. Discussion

At present, various RT-PCR-based commercial platforms approved by the Food and Drug Administration (FDA) are available for SARS-CoV-2 detection [21]. These platforms exhibit low LODs ranging from 8.26 to 250 copies/mL and up to 20 min analysis. While these technologies are promising, their colorimetric-based approaches do not facilitate the quantification of viral load within the sample. On the contrary, electrochemical approaches allow for quantification of viral load, which is important when monitoring patients with certain diseases.

### Biosensor Response

The biosensing platform used for the detection of viral nucleic acids in this study consists of two universal (target-independent) strands, namely USL and UMeB, and two target-specific strands, namely m and f. This modular biosensor design offers the advantage of standardizing the two most expensive components of the platform (USL and UMeB). As demonstrated in this work, a simple adjustment of the m and f strands to interrogate another nucleic acid target enabled the design of biosensors for the SARS-CoV-2 (genes S and N) and Influenza A virus, highlighting the potential for an affordable single biosensor capable of detecting multiple pathogens.

During the selection of a target fragment for the SARS-CoV-2 biosensor, the S gene was chosen due to its suitability for easy and reproducible amplification of SARS-CoV-2 RNA. Although the S-gene is susceptible to mutations, BLAST analysis reveals that the chosen region of the S-gene exhibits relatively low mutation rates (data not shown). Additionally, this platform can be designed to be tolerant to mutations by elongating the target-binding fragment of the m-strand.

The utilization of screen-printed gold electrodes (SPGEs) in conjunction with the 5S-4WJ platform opens up opportunities in the low-cost biosensor market [34], providing a sensitive and rapid detection of SARS-CoV-2. The low limit of quantification (LOQ) achieved by this platform, combined with the SPGE design, enables working with small sample volumes using a simple setup. The results presented here demonstrate that both gold disk electrodes (GDEs) and SPGEs exhibit low LOQ, at 0.57 and 0.92 nM, respectively, which can be further improved by increasing the hybridization time to reach the desired concentration levels relevant for the medical field.

As demonstrated here, when this system was coupled with an isothermal amplification method such as NASBA, down to 115 copies/µL of SARS-CoV-2 RNA could be detected with a corresponding NASBA/5S-4WJ platform on GDEs. This result is comparable to the average viral load in symptomatic patients (>103 copies/µL in pharyngeal samples after the first week of symptoms) [35], highlighting that this platform meets the requirements for early detection of the disease. However, it is worth noting that when applying this platform to real samples, variations in the limits of detection and quantification are expected. These discrepancies may arise due to preceding steps such as sampling and extraction, which can have an impact on the results. Nevertheless, these results demonstrate that once RNA is acquired, it can be successfully amplified using NASBA and subsequently detected by an electrochemical biosensor.

Crucially, we have demonstrated the versatility of the E-biosensor by showing its ability to detect both SARS-CoV-2 and Influenza A using the same GDE and two of the assay’s components (USL and UMeB). This ability to reuse the USL-modified electrode by simply washing it with deionized water and subsequently adding target-specific components for the hybridization step is noteworthy. This capability to easily adapt and reuse the biosensor for new targets, along with the ability to differentiate between viral targets, makes the reported sensing platform highly promising for affordable and accurate molecular diagnostics of viral diseases.

## 5. Conclusions

In summary, a five-stranded, four-way junction multi-purpose biosensor has been developed to detect the genetic signatures of SARS-CoV2 (genes N and S) and Influenza A (gene M) viruses. This biosensor utilizes two universal strands (USL and UMeB) and two target-specific strands (m and f) that can be easily adjusted for the detection of different pathogens while reusing the most expensive biosensor components: strands USL and UMeB. Notably, when combined with NASBA amplification, the biosensor was able to detect 115 copies/µL of SARS-CoV-2 RNA. The platform can be regenerated for new analyses with a simple washing step using deionized water, thus allowing a single electrode to be used for the detection of multiple viruses. The utilization of screen-printed gold electrodes (SPGEs) and a universal design for some biosensor components paves the way for the development of affordable electrochemical biosensors. Moreover, the combination of this biosensing platform with NASBA amplification provides a sensitive methodology that can be applied in real-world scenarios. By adhering to appropriate statistical validation procedures in line with FDA or ISO17025 guidelines, coupled with engineering refinements focused on enhancing sensitivity and affordability, this platform could be effectively deployed in low-income facilities.

## Data Availability

Not applicable.

## Supplementary Materials

The following supporting information can be downloaded at: https://www.mdpi.com/article/10.3390/bios13090874/s1, Table S1: Sequences of oligonucleotide used in the study; Figure S1: Response of the SARS-CoV-2 gene S sensor for a varied hybridization time (1, 5, 10, 15, 30, 45 and 60 minutes) using the SARS- S target (50 nM) on GDEs. Inset: SWV response before (black) and after (red) addition of the target with 10-min hybridization time; Figure S2: Calibration curves obtained for SARS-S-specific biosensor on SPGEs using varied con-centrations (5, 10, 25, 40, and 50 nM) of the SARS-S target with 10 min (black dots) and 30 min (red dots) hybridization time; Figure S3: Analysis of NASBA amplicons in 2% agarose gel. Samples containing ~2400, 824, 328, 115 or 39 copies/μL of SARS-Cov-2 RNA were subjected to NASBA reaction for 90 min. L: ssRNA ladder (the bands corresponding to 100- and 200-nt RNA fragments are labeled); NTC: NASBA no-template control. A grey box in the right gel image masks other samples that were analyzed in the same gel but are irrelevant for the reported work.
