# Supplementary material for: Cost-Effective Modular Biosensor for SARS-CoV-2 and Influenza A Detection
Source: Biosensors (Basel). 2023 Sep 7;13(9):874. doi: 10.3390/bios13090874 (PMC10526333; doi:10.3390/bios13090874)
Supplement: Supplementary file 1 [file biosensors-13-00874-s001.zip › biosensors-2563196-supplementary.pdf]

biosensors MDPI

Cost-Effective Modular Biosensor for SARS-CoV-2 and Influenza A Detection

Andrew Murray, Julio Ojeda, Omar El Merhebi, Percy Calvo-Marzal, Yulia Gerasimova and Karin Chumbimuni-Torres

Supporting Information

Table S1. Sequences of oligonucleotide used in the study.

Strand	Sequence (5′-3′)[1]
USL	/ThiolMC6-D/TTTTTTTTTTCGC**GTTAACATA**CAATAGATCGCG
UMeB	MeBIN/**CGGTACATTGTTGAG**[2]
SARS-S m-strand	**TTGAGCAATC**/iSp9/ **TATGTTAAC**TT**CTCAACAATGTACCG**[3]
SARS- S f-strand	**GATCTATTG**/iSp9/ **ATTTCATCTGTGAGCAAAGGTG**[3]
SARS -S	GTTTTGC**CACCTTTGCTCACAGATGAAAT**GATTGCTCAATACACTTCTGCACTGTTAGCG
SARS_FP	AATTCTAATACGACTCACTATAGGGAGAAGGGCCTTGGTGATATTGCTGCTAGA
SARS-RP	CGCTAACAGTGCAGAAGTGTA
SARS-S NASBA Amplicon	GGGAGAAGGGCCUUGGUGAUAUUGCUGCUAGAGACCUCAUUUGUGCACAAAAGUUU-AAC-GGCCUUACUGUUUUGC**CACCUUUGCUCACAGAUGAAAU**GAUUGCUCAAUACACUUCUGCACUGUUAGCG
SARS- N m-strand	**GCTGCCTGGAGTTGA**/Isp9/**TATGTTAAC**TT**CTCAACAATGTACCG**[3]
SARS- N f-strand	**GATCTATTG**/Isp9/ATTTCTTGAACTGTTGCGACTA[3]
SARS-N target	TAGTCGCAACAGTTCAAGAAATTCAACTCCAGGCAGCAGTAGGGGAACTTCTCCTGCTAG
InfA-M m-strand	**T CTC GGC TTT** /iSp9/ **TATGTTAAC**TT**CTCAACAATGTACCG**[3]
InfA-M f-strand	**GATCTATTG**/iSp9/ **GAGGGGGCCTGATGG AAC**[3]
InfA –M target	TATGTTCTCTCTATC**GTTCCATCAGGCCCCCTC**AAAGCCGAGATCGCGCAGAGACTTGAA

[1] Fragments of strands complementary to another are colored the same and/or underlined. [2]MeB: methylene blue. [3]iSp9: triethylene glycol spacer.

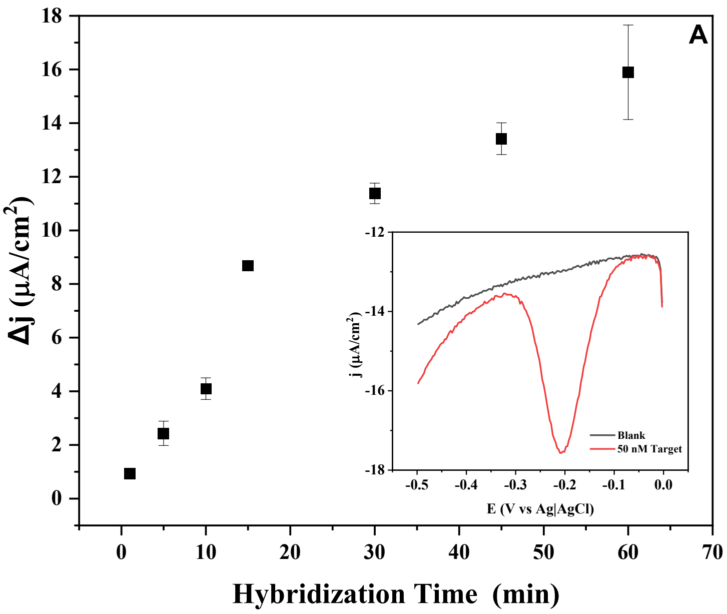

Figure S1. Response of the SARS-CoV-2 gene S sensor for a varied hybridization time (1, 5, 10, 15, 30, 45 and 60 minutes) using the SARS- S target (50 nM) on GDEs. Inset: SWV response before (black) and after (red) addition of the target with 10-min hybridization time.

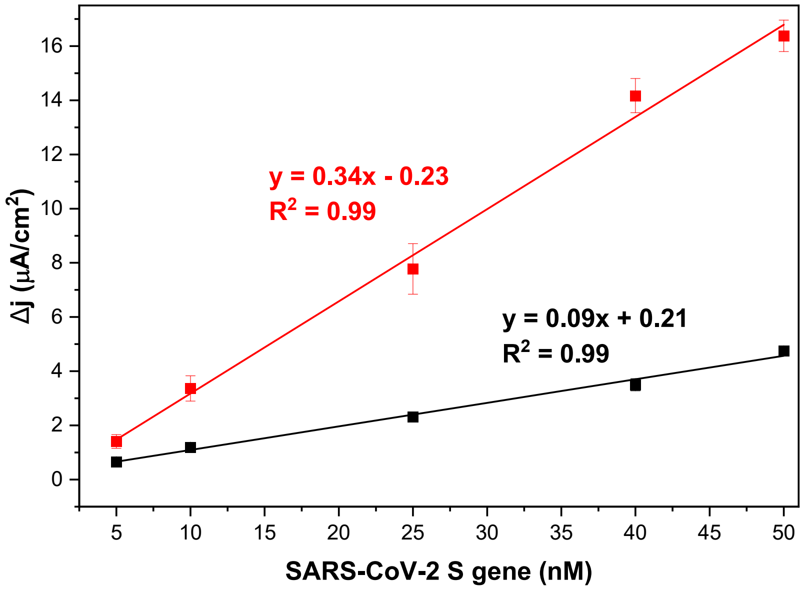

Figure S2. Calibration curves obtained for SARS-S-specific biosensor on SPGEs using varied concentrations (5, 10, 25, 40, and 50 nM) of the SARS-S target with 10 min (black dots) and 30 min (red dots) hybridization time.

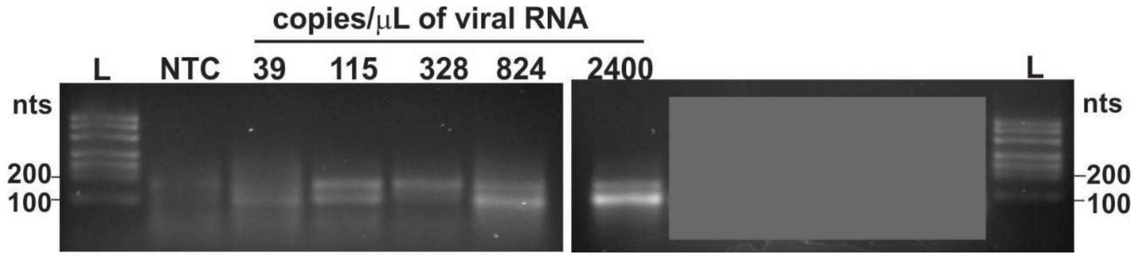

Figure S3. Analysis of NASBA amplicons in 2% agarose gel. Samples containing ~2400, 824, 328, 115 or 39 copies/uL of SARS-Cov-2 RNA were subjected to NASBA reaction for 90 min. L: ssRNA ladder (the bands corresponding to 100- and 200-nt RNA fragments are labeled); NTC: NASBA no-template control. A grey box in the right gel image masks other samples that were analyzed in the same gel but are irrelevant for the reported work.
